# Measuring the quality of life of long-term care service users in Japan: a cross-sectional questionnaire study

**DOI:** 10.1186/s12877-022-03662-8

**Published:** 2022-12-12

**Authors:** Koji Hara, Takayo Nakabe, Masayuki Tanaka, Yuichi Imanaka

**Affiliations:** 1 Department of Healthcare Economics and Quality Management, Graduate School of Medicine, Kyoto University, Yoshida Konoe-Cho, Sakyo-Ku, Kyoto City, 606-8501 Japan; 2 School of Economics and Business Administration, Yokohama City University, 22-2 Seto, Kanazawa, Yokohama, 236-0027 Japan; 3 The Database Center of the National University Hospitals, Tokyo Central Hospital North F8, The University of Tokyo Hospital, 7-3-1 Hongo, Bunkyo-Ku, Tokyo, 113-8655 Japan

**Keywords:** Quality of life, EQ-5D, WHO-5, Long-term care, Elderly, Care need level, Quality of service

## Abstract

**Background:**

In Japan’s super-aging society, the number of long-term care service providers is increasing, and the quality of care is a matter of concern. One aspect of the quality of care is the user’s quality of life. The questionnaires EQ-5D and WHO-5 are representative indicators of quality of life. Herein, we aimed to measure the quality of life in long-term care service users in Japan and to clarify the relationship between quality of life and the level of care required.

**Methods:**

A questionnaire study was conducted in 106 facilities of 22 corporations. In addition to the EQ-5D and WHO-5, sex, age, and the level of care required were assessed by descriptive statistics. Bonferroni’s multiple comparison test was used to analyze each quality of life score, and the differences by sex and age were analyzed multiple regression analyses, with each quality of life score as the objective variable.

**Results:**

Of 4647 cases collected, 2830 were analyzed, with no missing data. Both indicators tended to be lower than the general older population. Those scores tended to be higher in females than males (EQ-5D: males, 0.58 ± 0.26; females, 0.60 ± 0.24; *P* = 0.06 and WHO-5: males, 13.8 ± 5.92; females 14.9 ± 5.70; *P* < 0.001). In terms of age, those under 65 years old with specific diseases had lower EQ-5D scores than those in other age groups (*P* < 0.001); however, WHO-5 scores did not differ by age. Multiple regression analysis showed a significant association between the EQ-5D score and level of care required, except for support-required level 1, which tended to worsen as the level of care required increased. Conversely, the WHO-5 score was significantly lower for care need levels 2, 4, and 5.

**Conclusions:**

The quality of life of long-term care service users was worse than that of the general older population, it tended to be low among males and those under 65 years old with specific diseases. Furthermore, it gradually decreased as the level of care required increased. It is important to monitor users’ quality of life as a quality indicator of care, to improve and manage it.

## Background

Japan has the highest population of older people worldwide and has been implementing a long-term care (LTC) insurance system since 2000 [1]. The number of people requiring LTC services is increasing yearly, reaching 6.69 million or approximately 5.3% of the total population in 2020 [2]. According to a report by the Study Group on Elderly Care System for Future Supply and Demand of Long-term Care in the Ministry of Economy, Trade, and Industry, the number of people requiring LTC will peak in 2040, increasing to approximately 1.5 times the current level (roughly 9.88 million people) [3].

Japan’s LTC insurance system is based on “user-centeredness,” allowing users themselves to choose their desired services from various LTC service providers. Information on the quality of care is important for users to select appropriate care service providers and for the government to control the service quality of care service providers. However, no uniform way of measuring and disclosing the quality of care is currently available. Previous studies also emphasized the importance of quality of care for the benefit of the users, government monitoring and control, and promotion of the efforts of providers [4–6].

As people grow old, various chronic diseases develop, and their strength and muscle power gradually decline. Therefore, in LTC services, quality of life (QOL) becomes the primary goal rather than recovery from illness or improvement of physical functions. QOL means a sense of satisfaction with life and overall or subjective well-being, as influenced by health status, relationship with others, self-concept, and environment [7–9]. In fact, QOL is reportedly an indicator of quality of care [8, 10, 11]. Several QOL indicators have been developed and used in medical research as health-related QOL [12, 13]. Representative health-related QOL indices include the EQ-5D, SF-36, and WHO-5, and each has been used in various studies [8]. Among them, the EQ-5D and WHO-5 are known to be sensitive to QOL, especially physical and psychological QOL, with a limited number of questions (five questions for each indicator) and are successfully applied a wide range of study fields, including research on the elderly [14–17]. In addition, these measures have been used in many previous studies and can be easily compared with the results of this study. Therefore, they are considered appropriate for our target population.

When citizens of Japan are in need of LTC services because of a specific disease or old age, they have to be approved by the LTC approval board in their municipality before the level of care required is determined [1, 18, 19]. Users of LTC services must be 65 years old or older, but people between the ages of 40 and 64 can also avail of these services themselves if they suffer from a specific disease, such as cancer (terminal stage) or articular rheumatism, and need LTC for more than 6 months. Levels of care required are divided into two: support-required level (SL) and care need level (CL). SL indicates a lower level of care required than CL. SL is subdivided into two levels (1 and 2), whereas CL is subdivided into five levels (1, 2, 3, 4, and 5); the higher the number, the higher the level of care. Depending on the level of care required, different services and amounts of money are available for LTC insurance, and the level of care required is outlined in the Japanese LTC system. Therefore, it is important to evaluate the quality of care by the level of care required.

QOL is an important outcome for long-term care service users, but it is rarely measured in Japan, and the actual situation is not clear. To monitor and improve the quality of care, it is necessary to clarify the actual status of QOL indicators. From the large-scale data obtained in this study, we can present the reference QOL values based on the level of care required. This will be important data for quality monitoring by the government and LTC services providers, as well as for quality of care research by academia. Thus, we aimed to measure QOL in LTC service users in Japan and clarify the relationship between QOL indicators and the level of care required.

## Methods

### Questionnaire study

A self-administered, anonymous questionnaire survey was completed by LTC service users. The questionnaire included the EQ-5D and WHO-5 as QOL indicators and was assigned a unique ID for this study. The LTC staff distributed the questionnaires to the users and collected them after the users filled them out themselves. If the users could not answer the questionnaire by themselves due to physical reasons, family or staff listened to the user’s responses and answered on their behalf. Given the possibility of bias in case where the family or staff provided answers on behalf of the users, the answers were adjusted as dummy variables in the multivariate analysis. Information on the user's sex, age, and level of care required was collected from the staff separately from the user’s questionnaire. Using the unique ID for this study, the user’s information was matched to the data from the questionnaire.

### Setting

The questionnaires were distributed and collected in 106 facilities of 22 corporations in Japan between 2018 and 2020. The LTC service types included in-facility services (e.g., special nursing homes and housing for older adults with services), day services, and home-visit services. Users of day services are generally more independent than users of housing services. Given that more than half of the participating offices were in-facility services, this study did not conduct an analysis by service type but treats such types as adjustment variables.

### Outcomes

#### EQ-5D

The EQ-5D is a QOL indicator developed by EuroQol [20]. It consists of five items: mobility, self-care, usual activities, pain/discomfort, and anxiety/depression, rated on a five-level scale. This study used the Japanese version of the EQ-5D questionnaire, which was translated and confirmed for reliability and validity in Japanese by Shiroiwa et al. [21], with permission from EuroQol. The maximum value is 1, and the minimum value is − 0.025; a higher score corresponds with better QOL.

#### WHO-5

WHO-5 is a QOL indicator for mental health developed by the World Health Organization. It consists of five items (good spirits, calm and relaxed, active and vigorous, among them) that are measured in the positive dimension of mental health, each with a score from 0 to 5. The total score of this indicator can range from 0 (very poor) to 25 (very good). The survey used the Japanese version of the WHO-5 questionnaire, which is confirmed to be reliable and appropriate [22, 23]; higher score correspond with a better mental health status. A WHO-5 score of 13 or less is considered as a cutoff for poor mental health and is used to screen for depression. Previous studies have validated this cutoff value of the WHO-5 score with other indices (e.g., DSM-IV depression, CIDI ICD-10 depression, and CIDI suicidal) in various fields, including geriatrics, and reported high sensitivity and specificity [14, 24].

### Statistical analysis

Only those with complete data on the main outcome were examined. The main outcomes, which were based on EQ-5D and WHO-5 scores, were analyzed using descriptive statistics. Differences in the outcomes using the level of care required, sex, and age were evaluated by Kruskal–Wallis test. We used Bonferroni’s multiple comparison test to investigate the relationship between the QOL score and differences by sex and age. In contrast, we used a linear trend test of the mean value for each QOL score to determine the relationship between the QOL score and the level of care required. Moreover, we conducted multiple regression analysis, with the main outcomes as objective variables. The level of care required was used as the explanatory variable. As for the adjustment variables, sex, age, the type of LTC service, the corporation, and whether family or staff assistance was provided in the responses were used as dummy variables. All statistical data were analyzed using the statistical software R (ver. 4.1.2), with the significance level set at 5%.

## Results

There were 2830 cases out of 4647 who had complete data on the EQ-5D and WHO-5 as well as sex, age, the level of care required, the types of LTC services, and the corporation (The 1534 cases excluded were due to missing sex, age, and level of care required, whereas 283 cases ware excluded due to missing EQ-5D and WHO-5). The demographics of the respondents are shown in Table 1. Of these, approximately 67.1% were female and 32.9% were male, similar to the statistics on the percentage of people certified for LTC insurance nationwide (approximately 67.4% female and 32.6% male) [25]. Furthermore, the population percentages by required level of care, SL1 and SL2, are slightly lower than the national statistics, but the percentages for CL1-5 are nearly the same [25].

**Table 1:** Demographics of the respondents (number of people by sex, age, and level of care required)

				EQ-5D		WHO-5	
		N	%	Mean (± SD)	*P* value^1^	Mean (± SD)	*P* value^1^
SEX	Male	923	32.6%	0.58 (± 0.26)		13.8 (± 5.92)	
	Female	1907	67.4%	0.60 (± 0.24)	0.06	14.9 (± 5.70)	< 0.001
Age of users with specified diseases	< 65	126	4.5%	0.50 (± 0.26)		13.5 (± 6.35)	
	65–74	404	14.3%	0.60 (± 0.24)		14.3 (± 5.77)	
	75–84	1077	38.1%	0.60 (± 0.24)		14.5 (± 5.84)	
	> 84	1223	42.2%	0.59 (± 0.24)	< 0.001	14.7 (± 5.71)	0.134
Level of care required^2^	SL1	189	6.7%	0.70 (± 0.20)		16.1 (± 6.04)	
	SL2	273	9.6%	0.62 (± 0.20)		14.9 (± 5.73)	
	CL1	703	24.8%	0.67 (± 0.21)		15.2 (± 5.74)	
	CL2	632	22.3%	0.60 (± 0.23)		14.2 (± 5.64)	
	CL3	475	16.8%	0.57 (± 0.23)		14.5 (± 5.94)	
	CL4	362	12.8%	0.46 (± 0.24)		13.7 (± 5.48)	
	CL5	196	6.9%	0.37 (± 0.24)	< 0.001	12.9 (± 5.97)	< 0.001
Support for user responses by family and staff	User only	1008	35.6%	0.63 (± 0.24)		15.3 (± 6.06)	
	With family	331	11.7%	0.50 (± 0.23)		13.5 (± 5.73)	
	With staff	1491	52.8%	0.58 (± 0.23)	< 0.001	14.3 (± 5.55)	< 0.001

^1^ One-way ANOVA

^2^ *SL* Support-required level, *CL* Care need level

**Fig. 1:**
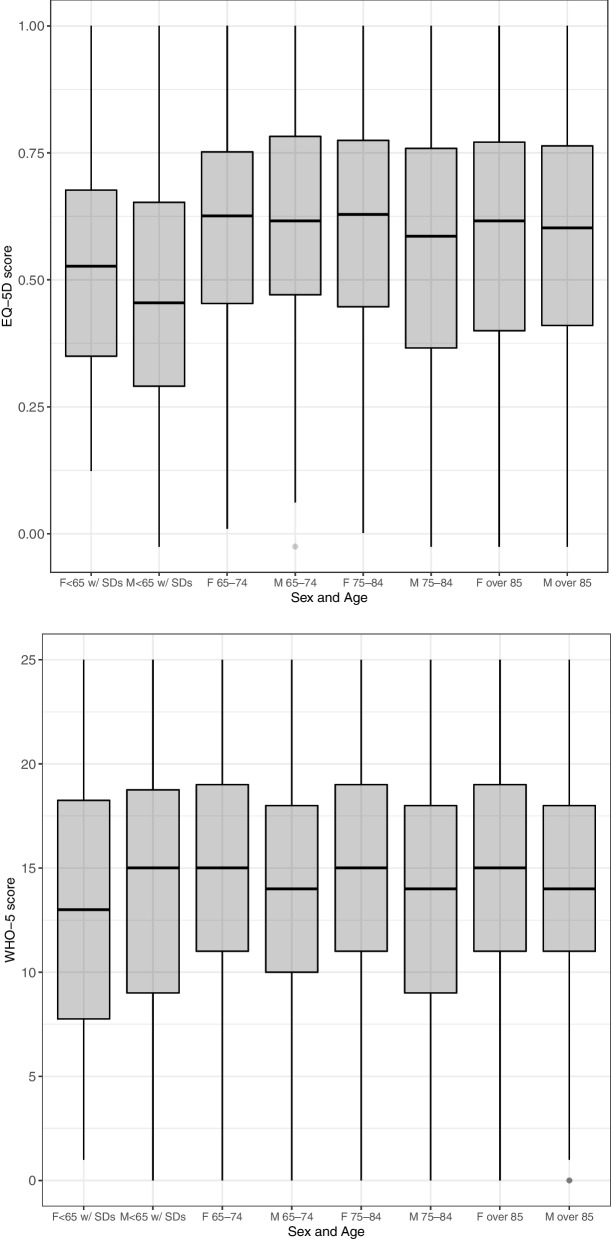
EQ-5D scores and WHO-5 scores by sex and age. ※ Bonferroni’s multiple comparison test. ※ F, female; M, male; SDs, specified diseases; SL, support-required level; CL, care need level. In EQ-5D scores, the score for males aged below 65 years with specific diseases was significantly lower than all categories except for the score for female aged below 65 years with specific diseases.. In WHO-5 scores, male aged 75–84 years had significantly lower scores than females aged 75–84 years and 85 years and older

**Fig. 2:**
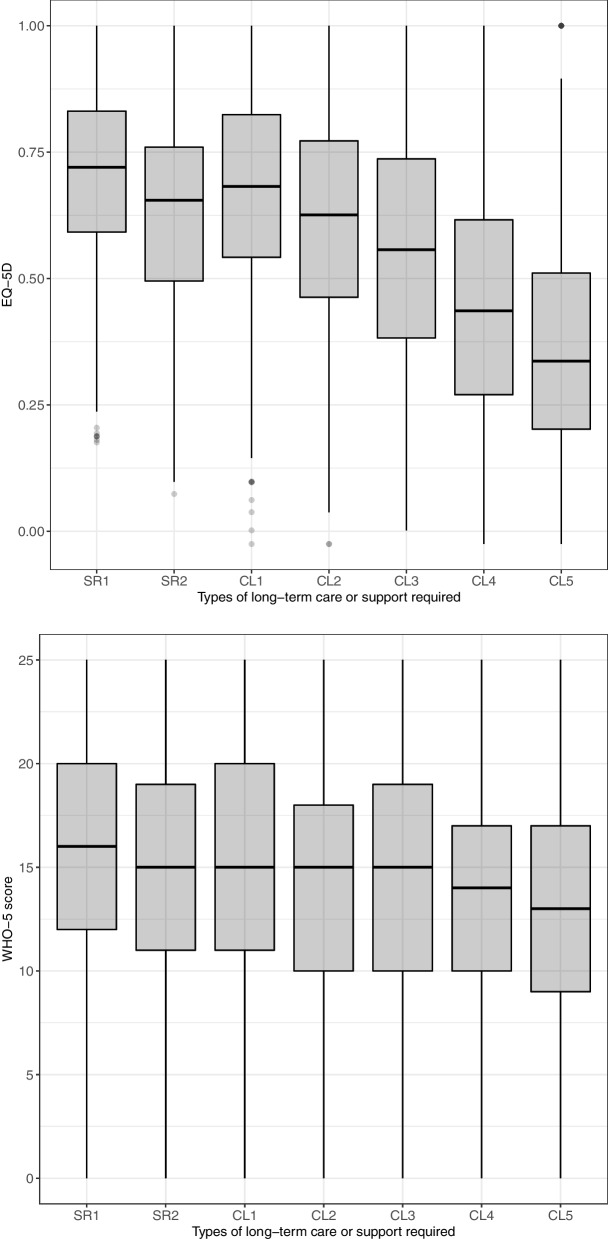
EQ-5D score and WHO-5 score by the level of care required. ※ SL, support-required level; CL, care need level. The results of the linear trend test between the EQ-5D score and the level of care required were significant (*P* < 0.001), similarly, those between the WHO-5 score and level of care required were significant (*P* < 0001)

Figures 1 and 2 show the box plots of EQ-5D and WHO-5 scores by sex, age, and level of care required. Scores on both the EQ-5D and WHO-5 tended to be higher in females than in males, especially on the WHO-5, with statistically significant differences (EQ-5D: males, 0.58 ± 0.26; females, 0.60 ± 0.24; *P* = 0.06 and WHO-5: males, 13.8 ± 5.92; females, 14.9 ± 5.70; *P* < 0.001). With regard to age, EQ-5D scores tended to be lower in those under 65 years old with specified diseases than in other age groups (*P* < 0.001); however, WHO-5 scores did not differ by age. The linear trend test results between the EQ-5D score and the level of care required were significant (*P* < 0.001); similarly, those between the WHO-5 score and level of care required were significant (*P* < 0001). The correlation coefficient between the EQ-5D and WHO-5 scores was 0.487 (*P* < 0.001; 95% CI, 0.458–0.514).

Table 2 shows the results of multiple regression analysis with the EQ-5D score as the objective variable. Regarding the level of care required, those who were CL5 had significantly lower EQ-5D scores (β =  − 0.29, *P* < 0.001), and clearly, EQ-5D scores tended to decrease as the level of care required increased. Table 3 shows the results of multiple regression analysis with the WHO-5 score as the objective variable. CL2, CL4, and CL5 tended to be significantly lower (CL2: β =  − 0.04, *P* = 0.05; CL4: β =  − 0.06, *P* = 0.01; CL5: β =  − 0.06, *P* < 0.001).

**Table 2:** Multiple regression analysis with EQ-5D score as the objective variable

		β	B	SE	*t* value	*P* value
Level of care required﻿^1^	SL1	0.01	0.01	0.02	0.43	0.67
	SL2	− 0.08	− 0.07	0.02	− 4.19	< 0.001
	CL1	Ref				
	CL2	− 0.10	− 0.06	0.01	− 4.69	< 0.001
	CL3	− 0.15	− 0.10	0.01	− 7.30	< 0.001
	CL4	− 0.27	− 0.19	0.01	− 12.83	< 0.001
	CL5	− 0.29	− 0.28	0.02	− 15.29	< 0.001

Dummy variables for sex, age, and type of service, the corporation, and whether family or staff assistance was provided in the responses were entered as adjustment variables

^1^ *SL* Support-required level, *CL* Care need level

**Table 3:** Multiple regression analysis with WHO-5 score as the objective variable

		β	B	SE	*t* value	*P* value
Level of care required^1^	SL1	0.02	0.39	0.46	0.84	0.40
	SL2	− 0.03	− 0.59	0.40	− 1.47	0.14
	CL1	Ref				
	CL2	− 0.04	− 0.60	0.31	− 1.96	0.05
	CL3	− 0.02	− 0.29	0.34	− 0.84	0.40
	CL4	− 0.06	− 097	0.38	− 2.52	0.01
	CL5	− 0.06	− 1.45	0.47	− 3.11	< 0.001

Dummy variables for sex, age, service type, the corporation, and whether family or staff assistance was provided in the responses were entered as adjustment variables

^1^* SL* Support-required level, *CL* Care need level

## Discussion

A large-scale QOL questionnaire study of LTC service users in Japan was done in this study. Both EQ-5D and WHO-5 scores tended to be higher females than males, especially in WHO-5, with statistically significant differences. In terms of age, those under 65 years old with specified diseases had lower EQ-5D scores than those in other age groups; however, WHO-5 scores did not differ by age. Both the EQ-5D and WHO-5 scores were significant in trend tests with the level of care required. However, multiple regression analysis with some adjustment variables showed that the EQ-5D score was associated with all care need levels except for SL1, and the WHO-5 score was associated with CL 2, 4, and 5.

Shiroiwa et al. (2016) reported that among Japanese people aged 70 years and older residing in the community, the EQ-5D score was 0.866 [21]. In our study, the EQ-5D score of LTC service users was 0.5–0.6, which is substantially lower than that reported in the previous study [21]. Similar to our findings, some studies have reported lower EQ-5D scores for users of LTC services, such as nursing homes, compared with independent home residents [26–28]. Eisele et al. (2015) noted that LTC service users, especially those in nursing homes, exercise at least once a week, which maintains their mobility and QOL [28]. Additional research is warranted to understand the factors affecting low scores and ways to maintain and improve them.

Those aged below 65 years with specified diseases showed significantly lower EQ-5D scores. In Japan, LTC insurance is usually available to people who are 65 years old or older and need LTC. People under 65 years old can also avail if they suffer from specific diseases, such as terminal cancer, Parkinson’s disease, and dementia. Users aged below 65 years with specified diseases tend to have a higher level of care required [1] and consequently, a lower EQ-5D score. Imai et al. (2008) measured the EQ-5D scores of day service users and found that females had higher scores than males and that the scores tended to decrease as the level of care required increased [29]. A similar trend was observed between the level of care required and EQ-5D scores in our study, but our EQ-5D scores showed no sex difference. This discrepancy may be explained by the fact that our data included not only day service users but also users of special nursing homes, housing for older adults with services, and fee-based nursing homes.

The WHO-5 score for older Japanese people is reportedly 16.6, 16.9, 16.7, and 14.8 for 65–69, 70–74, 75–79, and 80–84 years of age, respectively [30]. In our study, the LTC service users obtained mean WHO-5 scores of 13.5, 14.3, and 14.5 for those aged 64 and under with specific diseases, 65–74, and 75–84 years, respectively; therefore, they tend to have slightly lower WHO-5 scores than the general population. Thus, we need to monitor the mental health of LTC users and provide appropriate support and care.

According to a previous study, older females are more likely to have a lower mental health status than males because they have disadvantages in education, work, and income [31]. However, the female participants in our study had a tendency for significantly higher mental health status. Moreover, several papers have noted that older males are at higher risk for depression than older females, widowed men are more likely to be depressed than widowed women, and that females adapt to single life faster [32, 33]. Another study also reported more males than females with depressive symptoms among LTC facility users in Thailand, suggesting that depression may be related to social isolation [34]. Although more investigation is needed with regard to sex differences in mental health among the elderly, mental health care may be more important for males who use LTC services.

With regard to the relationship between WHO-5 scores and level of care required, those with CL2, CL4, and CL5 had lower WHO-5 scores. Similar to our findings, a study in Japan reported that the number of people with depressive symptoms increases as the level of care required increases, especially for those with CL 3, 4, and 5 [35]. Patra et al. (2017) reported that nursing home users are more likely to be depressed, especially those with depression-related factors, such as being bedridden and having fewer opportunities to go out [36]. Furthermore, most individuals with CL4 are dependent on a caregiver for communication and feeding, and most with CL5 require assistance with swallowing and have restricted joint movement [37]. Those with CL3 did not show a statistically significant association with WHO-5 scores, but we were unable to identify the reasons for this result in this study. One of the requirements for admission to a special nursing home in Japan is that the user must be of CL3 or higher, and those with CL3 often use in-facility services. However, the relationship between switching to in-facility services and mental health is unclear and requires further validation.

In general, a WHO-5 score of less than 13 is set as a cutoff for poor mental health [38]. In this study, 33.9% (958/2830) of the participants had a score of less than 13. A systematic review by Seitz et al. noted that 29% of users of LTC service had depressive symptoms [39]. Another study also reported that about 50% nursing home residents had depressive symptoms, and about 20% had major depression [40]. Therefore, the results of this study (approximately 30% LTC service users with poor mental health) are consistent with existing literature. Moreover, depression among older people is a health issue in many countries, including Japan [41, 42]. Maintaining and promoting mental health for LTC service users is a major challenge in a country with a growing elderly population.

We used the EQ-5D and WHO-5 as QOL indicators. These are validated indicators, including for the elderly, have reference values in many countries, and can be answered in a short time. Thereby, making them appropriate management indicators. In addition, this study allowed family and staff to assist users with the answering process. Responses involving family members and staff may have some bias. However, several studies have reported that responses by the care service users themselves and proxy responses by family members and staff were consistent to a certain degree [43, 44]. The study adjusted for this bias by using a dummy variable during multiple regression analysis to account for whether the respondents were assisted in their responses by family members or staff.

The following three implications can be derived from this study. First, the identification of the reference values for QOL indicators by the level of care required using large-scale data will enable quality monitoring and quality improvement by government and LTC service providers. Second, the results of the QOL indicators, WHO-5 and EQ-5D will provide important basic data for quality-of-care research and will accelerate quality-of-care research. This data can be expected to be useful not only in Japan but also in many other countries with containing aging populations. Finally, since the QOL of LTC service users is not better than that of the general elderly population, efforts to improve QOL and its evaluation are important. In the future, it will be necessary to consider including incentives and regulations for QOL in the LTC insurance system.

### Limitation

This study has several limitations. First, QOL by the type of care service was not clarified. To verify this point, researchers need to secure a larger sample size for each type of services. Second, although the corporation was adjusted as a dummy variable, the small number of corporations may have caused bias in the response results. However, since the study’s participants included both private and social welfare corporations, and the sample size was large, there was no substantial bias. Third, 40% of the collected questionnaires in this study had missing data and were excluded from the analysis (1817 cases out of 4647). This is attributable to the insufficiency with regard to the collection of information on the users’ sex, age, and level of care as well as cases of mismatched user IDs. Finally, considering the influence of the Japanese LTC insurance system, generalizing results to other countries is difficult.

## Conclusion

The QOL of LTC service users in Japan was worse than that of the general older population, according to this large-scale questionnaire study. In particular, QOL tended to be low among males and those younger than 65 years with specified diseases. In addition, QOL gradually decreased as the level of care required increased. It is important to monitor users’ QOL as a quality indicator of care, in order to improve and manage it.

## Data Availability

The data sets generated and analyzed during the current study are not publicly available due to concerns about the data privacy of the study participants, but de-identified data are available from the corresponding authors upon reasonable request.

## Abbreviations

LTC: Long-term care
QOL: Quality of life
CL: Care need level
SL: Support-required level
